# Supplementary material for: FLIMJ: An open-source ImageJ toolkit for fluorescence lifetime image data analysis
Source: PLoS One. 2020 Dec 30;15(12):e0238327. doi: 10.1371/journal.pone.0238327 (PMC7773231; doi:10.1371/journal.pone.0238327)
Supplement: Attachment — Submitted filename: FLIMJ Review.pdf [file pone.0238327.s001.pdf]

FLIMJ Review

The authors present a report of their validation and use cases for an important open source software tool, FLIMJ. The work is important because there are not many easy-to-use lifetime image analysis tools integrated with broader image analysis tools such as those that can be found in the FIJI ecosystem.

While it does not appear the authors have developed novel analysis algorithms (the toolkit is built using the previously existing FLIMLib library and Scijava components) or answered an original research question using FLIMJ, the reviewer believes that the transformation of the FLIMLib backend into a user-friendly tool is of significant relevance to the PLOS One audience, not to mention an endeavor requiring significant investment of time and expertise. The combined result is something new.

The article requires significant work to make it more readable and compelling.

I recommend the article be accepted for publication after the authors address the following items:

1. **Technical comments.** The manuscript is technically sound, but I have the following comments.
    a. Fig. 1 should be arranged so that the position and/or shape of the items helps to reinforce the relationships implied by the arrows. If different colors are used, their significance should be enumerated in the figure legend. Stratifying the items along one spatial dimension to reflect back-end to front-end components could also be helpful. As is, non-developers (probably the majority of PLOS One readers) will not benefit much from a jumble of similar sounding bubbles with somewhat overwhelming arrows. More thought could help make the structure and significance of the components more readily apparent.
    b. I think the sentence “However, more connectors can be added to make the library accessible to many high-level programmers using Python…” is referring to doing this by way of SWIG framework, but it is not clear. Please clarify if there is another tool the authors think would be better for other language interfaces.
    c. Is there a reason R’s site is linked and not Python’s?
    d. In the Methods section describing FLIMLib’s LM nonlinear least squares fitting, please elaborate more on how the noise model optimizes performance.
    e. In the Methods section describing FLIMLib’s Bayesian Inference implementation, it’s unclear which in vitro experiments are mentioned, though it seems likely to be ref 27. If so, moving the in-text citation to the same sentence mentioning the experiments would make this more clear.
    f. As I write this review, I’m noticing that FLIMJ is not listed on the update sites https://imagej.github.io/list-of-update-sites/, though the preprint is out. I was expecting to be able to try it based on the conversation here on imagesc

https://forum.image.sc/t/flimj-slimcurve-upate-site/31376/3. What is the reason for the missing update site? Is there some compatibility issue with current FIJI build? https://forum.image.sc/t/coba-imagej-fiji-summer-2020-roadmap/39453. When will the Plug-in be available? It seems if there is an issue here it should be addressed before the publication of the new tool occurs. UPDATE: I found a usable update site address here https://github.com/flimlib/flimj-ui when I went looking for the example notebooks (FLIMJ Ops repo) mentioned in the article. There is mention of two notebooks, but I only see one (9/7).

g. Fig. 2 should present the analysis pipeline first, then the processing results, as in Fig. 3.
h. In Fig. 2, the segmentation classes/labels should be indicated for ease of interpretation of the figure. It's not very clear what "FLIMJ pseudocolor output" is referring to. The text mentions the Figure shows calculated FRET scores (?)… but I only see one efficiency given in the legend. It would also make sense, given the audience, to elaborate on what a FRET efficiency of 3.1% indicates.
i. The text in the Results section describing the segmentation pipeline is not clear and the description in the legend is too cursory. It would make the article more clear to expand the description of the pipeline in text.
j. The imaging details are somewhat odd appearing in the Segmentation use case Results section. Consider moving them to the methods or letting the reference to the study stand alone. In contrast, the details of imaging for the Colocalization use case appear to be missing. Please clarify if these images are from the same or different instrumentation.
k. Fig. 3 Panel B needs to be replaced with a higher resolution image so the audience can read the GUI text. Legend should describe the parameters in the software panel.
l. Fig. 3 Panel C should have a scale bar and indicate what the red and green channels are representing on or near the image.
m. I need a little more help understanding the last sentence of Use Case II: Colocalization in the Results that says "This colocalization can help us find unwanted GFP signal bleed through to the NADH channel and help optimize…" The whole section is a bit unclear. What I gather is that it's desirable to identify cell types molecularly (fluorescent protein reporter, immunohistochemistry) and get to also acquire FLIM data to understand the metabolic state of the population of interest. However, markers with emissions overlapping the metabolic species of interest for FLIM imaging will interfere with the lifetime estimate. I'm failing to connect how the FLIMJ analysis addresses this bleedthrough problem. Please rework this section for clarity.
n. Please describe how the single-component validation data are simulated and how they reflect conditions different from "low photon images such as auto-fluorescence-based FLIM and similar low-light FLIM experiments."
o. Is it possible to provide some other validation for fitBayes? Why does it perform poorly as lifetime increases?

   p. Fig 5. Should lead with the ground truth image and follow with the fit comparisons and the one no-fit phasor representation.
   q. If possible, can the authors provide template macros/scripts/notebooks for the use cases presented at the referred github repo (or if they exist, point to them a little more clearly)?
   r. In the third paragraph of the discussion, the transition from the first to second paragraph doesn't make sense. Perhaps some lines are missing. The whole paragraph is pretty fluffy.
   s. Add panel labels for Fig. 6 and improve the figure legend so it matches the terminology in the figure itself (A1… A2…).
2. **Structure.** Starting with the validation section and progressing to the use cases is a more logical progression for the report.
3. **Writing Style.** The writing style needs considerable improvement to meet the standard for a scientific article.
   a. In the abstract, the third sentence should be revised. "FLIM allows for the measurement of how long a fluorophore stays in an excited energy state and is affected by changes in its chemical microenvironment, such as proximity to other fluorophores, pH, and hydrophobic regions." The second clause reads as applying to FLIM itself, but it is more accurate to apply it to the measurement by changing the bold text to "and this measurement is"
   b. In the abstract, the authors write that "FLIMJ offers FLIM fitting routines with seamless integration with other ImageJ components". It seems appropriate to qualify this statement by using "with many other" ImageJ components or "with most other" ImageJ components, to alert the reader of the more nuanced ImageJ ecosystem.
   c. In the first paragraph of the introduction, the third, fourth, and fifth sentences are too loose and should be combined into one concise statement of the important measurements/insights that can be gleaned from FLIM.
   d. In the first sentence of the second paragraph of the introduction, "While more is needed" need a noun as it is awkward to wait for "advances" to appear (the presumed noun). "While more work is needed" would be better.
   e. In the fifth sentence of the second paragraph of the introduction, "...there are new developments for new turnkey open analysis FLIM software tools such as FLIM fit from the Paul French's group." the second "new" should be removed and "the" in front of "Paul French" should be removed.
   f. In the third paragraph of the Introduction, after the summarized points the reader is directed to (see below). It's not clear what "see below" is referring to.
   g. In the first paragraph of the Methods, the use of the word "consumes" is awkward.
   h. In the first paragraph of the FLIMLib section of the Methods, the second sentence says, "...the library can be compiled to run fast as a native executable on Linux, Windows, or MacOS." "fast" is an adjective but is used as an adverb. Remove "fast."

i. In the Results section under Use Case I: Segmentation, the first paragraph should probably have more citations or move the in text citations for refs 30,35 to earlier.
j. In the Results section under Use Case I: Segmentation, in the fifth paragraph it would make the sentence more clear to replace "... to help identify species." with "...to help identify dimer species."
k. Ensure all Figures are referred to in the text.
l. In the second paragraph of the Discussion, the first and second and third sentences should be joined so the second and third sentences are not incomplete. The subsequent sentence is very confusing. Are there references that could be cited to make it more clear what the authors are referring to?

4. **Typos.**
    a. In the first paragraph of the FLIMLib section of the Methods, the link for the flimlib github site should have a period after it, and there seem to be too many spaces in front of it.
    b. In the Methods section on Ops API, "While fully preserving FLIMLib's granularity of control overfitting..." should be "... over fitting…"
    c. In the Notebooks for FLIM Analysis section of the Methods, in the first sentence is awkward. Consider changing "The notebooks are a recent addition to the scientific processing scenario that helps one…" to "Notebooks are a recently developed scientific programming tool that help one…" or something like that.
    d. In the Fig. 2 legend, there is an extra parenthesis just before "B)".
    e. Refs 41, 42, and 45 need a space in front of their in-text citations.
    f. In Fig. 5, there should be a space in "10ns".
    g. In the first paragraph discussing the two-component validation, "1-comp" should be "1-component."
    h. In the first paragraph discussing the two-component validation, there should be a space in "4ns."
