# Supplementary material for: FLIMJ: An open-source ImageJ toolkit for fluorescence lifetime image data analysis
Source: PLoS One. 2020 Dec 30;15(12):e0238327. doi: 10.1371/journal.pone.0238327 (PMC7773231; doi:10.1371/journal.pone.0238327)
Supplement: Attachment — Submitted filename: FLIMJ_ResponseToReviewers.docx [file pone.0238327.s002.docx]

Dear Editor,

First of all, we would like to thank the reviewers for the detailed comments and suggestions to improve the manuscript. We have made major revisions following the reviewers' recommendations and advice. We have modified the figures, fixed language, and added additional data to validate the claims on the analysis. Some of the comments, especially on extending the analysis out of the ImageJ was not pursued as that additional work would fall out of the scope of this work. With these additions, we hope our work demonstrating new FLIM Analysis tools for ImageJ would be suitable for publication.

We have detailed our answers to the comments below the reviewer's comment in blue ink. A marked and unmarked copy of the manuscript is also provided.

-Kevin Eliceiri and all authors

## Reviewer #1:

The article requires significant work to make it more readable and compelling. I recommend the article be accepted for publication after the authors address the following items:

We want to thank the reviewer for the valuable comments on our manuscript. We really appreciate your time spent to improve our manuscript and sincerely thank you for all your efforts. Replies to all the comments are listed below in blue ink. Changes in figures are directly changed in the final manuscript.

### 1. Technical comments.

The manuscript is technically sound, but I have the following comments.

A. Fig. 1 should be arranged so that the position and/or shape of the items helps to reinforce the relationships implied by the arrows. If different colors are used, their significance should be enumerated in the figure legend. Stratifying the items along one spatial dimension to reflect back-end to front-end components could also be helpful. As is, non-developers (probably the majority of PLOS One readers) will not benefit much from a jumble of similar-sounding bubbles with somewhat overwhelming arrows. More thought could help make the structure and significance of the components more readily apparent.
   Thank you for the suggestion. We have simplified the Figure as suggested in the article.
B. I think the sentence "However, more connectors can be added to make the library accessible to many high-level programmers using Python…" is referring to doing this by way of SWIG framework, but it is not clear. Please clarify if there is another tool the authors think would be better for other language interfaces.

   Thank you for the suggestion; we have made the necessary changes. SWIG, Cython and similar extension tools offer the capability of exposing the FLIMLib functions to Python and other high-level languages. Although this work is doable and potentially useful, these extensions are not in the scope of our work. This work is solely about FLIMJ, i.e. the integration of FLIMLib to ImageJ, and not directly into other languages. We do demonstrate usage of FLIMLib and FLIMJ from Python via the PyImageJ bridge, which exposes all of ImageJ to Python in a convenient way.
C. Is there a reason R's site is linked and not Python's?

   -We have now included Python and linked the python website.
D. In the Methods section describing FLIMLib's LM nonlinear least squares fitting, please elaborate more on how the noise model optimizes performance.

   The noise models were earlier discussed in Rowley et al and readers are directed to that work. We have added these two sentences to the text.
   "Possible noise models include Maximum Likelihood Estimation: Poisson noise model (30) and Gaussian: Variance for Gaussian distribution (Least-squares fitting). These statistical noise models used here were described in detail in Rowley's work (27)."
E. In the Methods section describing FLIMLib's Bayesian Inference implementation, it's unclear which in-vitro experiments are mentioned, though it seems likely to be ref 27. If so, moving the in-text citation to the same sentence mentioning the experiments would make this more clear.

   Yes. Thank you for pointing out this mistake. We have moved the citation to match the text.
F. As I write this review, I'm noticing that FLIMJ is not listed on the update sites <https://imagej.github.io/list-of-update-sites/>, though the preprint is out. I was expecting to be able to try it based on the conversation here on imagesc <https://forum.image.sc/t/flimj-slimcurve-upate-site/31376/3>.
   a. What is the reason for the missing update site? Is there some compatibility issue with current FIJI build?
   b. When will the Plug-in be available?
   c. It seems if there is an issue here it should be addressed before the publication of the new tool occurs. UPDATE: I found a usable update site address here <https://github.com/flimlib/flimj-ui> when I went looking for the example notebooks (FLIMJ Ops repo) mentioned in the article. There is mention of two notebooks, but I only see one (9/7).

a) This was just an unintentional mistake from our side and no build-compatibility issues. We have rectified this issue of the missing FLIMJ update-site link in the ImageJ update sites menu.
b) The Plug-in is available on the update-site list from Oct15 and FIJI users can download the plugin at ease.
c) We apologize for the confusion and changes in the Git repository. The second notebook is a python-notebook that uses PyimageJ and is available in the *flimj-ops/tree/python_notebook* branch. However, there has been some restructuring in PyImageJ and this ipython-notebook is currently limited to work with pyjnius version 1.2.0. This is why users could not locate this notebook in the master branch. (For more details, please refer <https://github.com/flimlib/flimj-ops/pull/4>). This branch will be integrated to the flimj-ops as soon as the dependency tree is cleared.

G. Fig. 2 should present the analysis pipeline first, then the processing results, as in Fig. 3.

   Thank you for the suggestion. We have restructured the panels to match the reviewer's suggestion.
H. In Fig. 2, the segmentation classes/labels should be indicated for ease of interpretation of the Figure. It's not very clear what "FLIMJ pseudocolor output" is referring to. The text mentions the Figure shows calculated FRET scores (?)… but I only see one efficiency given in the legend. It would also make sense, given the audience, to elaborate on what a FRET efficiency of 3.1% indicates.

   We apologize for the muddled caption and have corrected the mismatch. The caption has been replaced with a new one that omits "FRET score" and removes "pseudo-color" from the Figure.
I. The text in the Results section describing the segmentation pipeline is not clear and the description in the legend is too cursory. It would make the article more clear to expand the description of the pipeline in text.

   Thank you for pointing out the obscurity. We have added a descriptive pipeline in the Use-case 1 section.
J. The imaging details are somewhat odd appearing in the Segmentation use case Results section. Consider moving them to the methods or letting the reference to the study stand alone. In contrast, the details of imaging for the Colocalization use case appear to be missing. Please clarify if these images are from the same or different instrumentation.

   Thank you for the suggestion. Figures 2 and 3 are collected independently using separate instruments and are presented as use-case scenarios of the fitting. We have included a few sentences on how colocalization instrumentation was implemented and cited the article.
K. Fig. 3 Panel B needs to be replaced with a higher resolution image so the audience can read the GUI text. Legend should describe the parameters in the software panel.

   Thank you for the suggestion. We have replaced figure3 panel B with a higher resolution picture and included the parameters in the legend. However, this is a community-driven project, and we expect the user interface to evolve over time.
L. Fig. 3 Panel C should have a scale bar and indicate what the red and green channels are representing on or near the image.

   Thank you for pointing this out to us. We have corrected figure3 panel C with inset markers, scale bars, and legends.
M. I need a little more help understanding the last sentence of Use Case II: Colocalization in the Results that says "This colocalization can help us find unwanted GFP signal bleed through to the NADH channel and help optimize…" The whole section is a bit unclear. What I gather is that it's desirable to identify cell types molecularly (fluorescent protein reporter, immunohistochemistry) and get to also acquire FLIM data to understand the metabolic state of the population of interest. However, markers with emissions overlapping the metabolic species of interest for FLIM imaging will interfere with the lifetime estimate. I'm failing to connect how the FLIMJ analysis addresses this bleedthrough problem. Please rework this section for clarity.

   We apologize for the confusion; the goal is not to address GFP bleedthrough to the NADH channel but to use the GFP signal as a colocalization marker for NADH targets. We have removed the confusing statements. This use-case has been recently published, and a citation to this work has been added to the manuscript for the readers.	<https://www.frontiersin.org/articles/10.3389/fnins.2020.00931/full>
N. Please describe how the single-component validation data are simulated and how they reflect conditions different from "low photon images such as auto-fluorescence-based FLIM and similar low-light FLIM experiments."

   The single-component validation data does not have any noise model. The data was simulated as an exponential decay convolved with a gaussian IRF of fixed (0.2ns) width. We have updated figure 5 to represent the text better: including low-light conditions.
O. Is it possible to provide some other validation for fitBayes? Why does it perform poorly as lifetime increases?

   Thank you for the suggestion. We examined the methods more meticulously, and we found some artifacts present in the automated fitting steps. The simulation data artificially raised the offset (Z) parameter of the fit for Bayesian. This is because the Bayesian fits use the data outside cursor positions (unlike other fits), and it is difficult to automate the fitting process for a large range. Hence we removed this panel comparing Bayesian fit with ground truth. Instead, we added three panels comparing how Bayesian fit gives more reliable data in the presence of varying noise levels.

   The bar graph below shows the decreasing standard deviation between the difference of fit lifetime-values of clean and noisy data with increasing percent of photons from the clean curve. This bar graph is not presented in the manuscript but only provided for the reviewer's perusal. (this plot shows the standard deviation of the data shown in the new panel Fig5-F)

P. Fig 5. Should lead with the ground truth image and follow with the fit comparisons and the one no-fit phasor representation.

   Thank you for the suggestion. We have changed Figure 5 panels according to the reviewer's recommendation.
Q. If possible, can the authors provide template macros/scripts/notebooks for the use cases presented at the referred github repo (or if they exist, point to them a little more clearly)?

   Thank you for the suggestion. Unfortunately, the use-cases (colocalization and segmentation) relied entirely on GUI operations, so no scripts/macros could be added. But, we have added a list of general macros and groovy one-liners to the repository after submitting the manuscript intending to help users jump-start the FLIMJ applications. This list is continuously refining based on community effort.
   Please see: https://github.com/flimlib/flimj-ops/blob/master/groovy.md
R. In the third paragraph of the discussion, the transition from the first to second paragraph doesn't make sense. Perhaps some lines are missing. The whole paragraph is pretty fluffy.

Thank you for this; we have edited this section to improve readability.

S. Add panel labels for Fig. 6 and improve the figure legend so it matches the terminology in the Figure itself (A1… A2…).

   Thank you for pointing out the missing panel labels. We have updated the Figure with labels and changed the caption following the reviewer's suggestion.

### 2. Structure.

- Starting with the validation section and progressing to the use cases is a more logical progression for the report.
  This is an excellent suggestion; however, we want to highlight the FLIM operations in Fiji rather than the prowess of our fitting library. We present this work as a user-friendly Fiji fitting application. The current structuring is only demonstrating the focus-group. The rigor and analysis validation is not our primary focus but will be a goal for future efforts.

### 3. Writing Style.

- The writing style needs considerable improvement to meet the standard for a scientific article.
  We have fixed wording and grammar errors pointed out by the reviewer and some others during proofreading. The authors thank the reviewer for the detailed read.

A. In the abstract, the third sentence should be revised. "FLIM allows for the measurement of how long a fluorophore stays in an excited energy state and is affected by changes in its chemical microenvironment, such as proximity to other fluorophores, pH, and hydrophobic regions." The second clause reads as applying to FLIM itself, but it is more accurate to apply it to the measurement by changing the bold text to "and this measurement is"
   **⊠** Fixed the sentence exactly as the reviewer suggested
B. In the abstract, the authors write that "FLIMJ offers FLIM fitting routines with seamless integration with other ImageJ components". It seems appropriate to qualify this statement by using "with many other" ImageJ components or "with most other" ImageJ components, to alert the reader of the more nuanced ImageJ ecosystem.
   **⊠** Fixed the sentence exactly as the reviewer suggested
C. In the first paragraph of the introduction, the third, fourth, and fifth sentences are too loose and should be combined into one concise statement of the important measurements/insights that can be gleaned from FLIM.

   Thank you, we have done this.
D. In the first sentence of the second paragraph of the introduction, "While more is needed" need a noun as it is awkward to wait for "advances" to appear (the presumed noun). "While more work is needed" would be better.
   **⊠** Fixed the sentence exactly as the reviewer suggested
E. In the fifth sentence of the second paragraph of the introduction, "...there are new developments for new turnkey open analysis FLIM software tools such as FLIM fit from the Paul French's group." the second "new" should be removed and "the" in front of "Paul French" should be removed.
   **⊠** Fixed the sentence exactly as the reviewer suggested
F. In the third paragraph of the Introduction, after the summarized points the reader is directed to (see below). It's not clear what "see below" is referring to.
   **⊠** we have removed the unclear link text "see below ".
G. In the first paragraph of the Methods, the use of the word "consumes" is awkward. **⊠** We have fixed the mistake. Replaced 'consumes' with 'uses'
H. In the first paragraph of the FLIMLib section of the Methods, the second sentence says, "...the library can be compiled to run fast as a native executable on Linux, Windows, or MacOS." "fast" is an adjective but is used as an adverb. Remove "fast." **⊠** Fixed the sentence exactly as the reviewer suggested
I. In the Results section under Use Case I: Segmentation, the first paragraph should probably have more citations or move the in text citations for refs 30,35 to earlier. **⊠** Fixed the sentence exactly as the reviewer suggested
   In the Results section under Use Case I: Segmentation, in the fifth paragraph it would make the sentence more clear to replace "... to help identify species." with "...to help identify dimer species."
   **⊠** Fixed the sentence exactly as the reviewer suggested
J. Ensure all Figures are referred to in the text.
   **⊠** We verified all figures are mentioned in the text. Figure 1 reference was added.
K. In the second paragraph of the Discussion, the first and second and third sentences should be joined so the second and third sentences are not incomplete. The subsequent sentence is very confusing. Are there references that could be cited to make it more clear what the authors are referring to?
   Thank you for this; we have improved the wording in this paragraph now.

### 4. Typos.

A. In the first paragraph of the FLIMLib section of the Methods, the link for the flimlib github site should have a period after it, and there seem to be too many spaces in front of it.
   **⊠** Fixed the sentence exactly as the reviewer suggested. We did not identify the extra spaces but removed all potential errors during pdf conversion.
B. In the Methods section on Ops API, "While fully preserving FLIMLib's granularity of control overfitting..." should be "... over fitting…"
   **⊠** Fixed the sentence exactly as the reviewer suggested.
C. In the Notebooks for FLIM Analysis section of the Methods, in the first sentence is awkward. Consider changing "The notebooks are a recent addition to the scientific processing scenario that helps one…" to "Notebooks are a recently developed scientific programming tool that help one…" or something like that.
   **⊠** Fixed the sentence exactly as the reviewer suggested. "Notebooks are scientific programming tools that bind the processing pipeline (codes) with the input and output assisted with detailed comments using markdown-style notes."
D. In the Fig. 2 legend, there is an extra parenthesis just before "B)".
   **⊠** Fixed the sentence exactly as the reviewer suggested.
E. Refs 41, 42, and 45 need a space in front of their in-text citations.
   **⊠** Fixed the sentence exactly as the reviewer suggested. Verified all references and parentheses usage.
F. In Fig. 5, there should be a space in "10ns".
   **⊠** Fixed the sentence exactly as the reviewer suggested. Verified and fixed all units used.
G. In the first paragraph discussing the two-component validation, "1-comp" should be "1-component."
   **⊠** Fixed the sentence exactly as the reviewer suggested.
H. In the first paragraph discussing the two-component validation, there should be a space in "4ns."
   **⊠** Fixed the sentence exactly as the reviewer suggested.

## Reviewer #2:

The scientific community using FLIM may potentially be benefited by using this free access toolkit. Below there are some comments that might help to improve the publication of this manuscript.

Thank you for the detailed review and valuable suggestions. We have made changes according to your recommendations. Please see the listed replies to all the comments below.

1. The proposed FLIM tool kit will greatly benefit those whose research is based on analysis of FLIM features and not on development of methods for FLIM analysis. However, there is a community of researches using machine learning libraries on FLIM data that are not available for ImageJ such as the MATLAB Machine learning toolbox, TensorFlow, and PyTorch - to mention some. Currently, Python is gaining popularity through the microscopy community due to the vast availability of scientific toolboxes. The authors stated that FLIMJ can be integrated into Python.
   However, a Jupyter notebook that accesses ImageJ ops through the PyImageJ interface to invoke FLIMJ ops is required. It would be also very beneficial to have a direct FLIMLib library for Python that can be directly installed via Conda or PIP to straightforwardly connect it with the statistical and machine learning libraries for Python without interfacing PyImageJ.

   This suggestion is entirely in agreement with our approach of FLIMJ as an extensible scientific tool. We have a python notebook in the flimj-ops doing exactly what the reviewer pointed out as a necessary step. However, the notebook is pending a pull-request (on flimj-ops repository) because of certain Pyimagej dependency fixes. The notebook is working and reviewable at <https://github.com/flimlib/flimj-ops/blob/python_notebook/notebooks/FlimLib_from_python.ipynb>
2. ImageJ can read different image file formats such as TIFF, NIfTI, DICOM, HDF5, etc. However, to my knowledge, it cannot read proprietary file formats of commercial FLIM software such as Becker & Hickl (B&H) and PicoQuant. Does FLIMJ provide integration of file formats from commercial FLIM systems?

   ImageJ2 and Fiji do support several FLIM file formats, both proprietary and open. As such, FLIMJ does not implement support for lifetime file formats. Rather, it is separately handled by ImageJ I/O plugins, backed by the SCIFIO library (https://scif.io/). The base version of ImageJ2 includes a SCIFIO plugin for the open Image Cytometry Standard (ICS) format, which the UCL Cancer Institute uses to store lifetime data, as well as a plugin for the Becker & Hickl SDT format. Further, the Fiji distribution of ImageJ includes Bio-Formats, which adds support for additional lifetime image formats, including Becker & Hickl .spc, PicoQuant .bin, Lambert .fli, and others (https://docs.openmicroscopy.org/bio-formats/latest/supported-formats.html). There are also numerous open-sourced community efforts to read proprietary files available such as ptu-reader (<https://github.com/ekatrukha/PTU_Reader>).
3. Could the authors please talk about the reproducibility of image processing from software of commercial FLIM systems, since commercial software uses proprietary algorithms for decay fitting and image segmentation that might differ from the ones provided by FLIMJ?

   The fitting libraries for most commercial FLIM systems are proprietary, and we can only make a blind-test comparison. We have compared the results between commercial fitting provided by B&H (SPCImage), and we found no loss in resolution or precision in our tests. This comparison is described in section 3 of the results. However, we realize that this is another avenue of improvement because FLIMLib is an open-sourced, community-powered research-friendly platform that can gain a larger user-interest than its commercial alternatives.
4. Some research studies require analysis of 3D volumetric FLIM images in time (4D imaging), and in some cases longitudinal multispectral volumetric FLIM imaging (5D imaging). Could the authors please discuss how FLIMJ would provide support these kind of data without using programming since the idea of using ImageJ is to avoid creating your own image processing scripts?

   ImageJ targets a broad spectrum of use cases, from fully GUI-based workflows with no programming or scripting to fully automated and reproducible analysis using scripts, and a range of uses in between these two extremes. With FLIMJ, we have tried to cater to uses along this full spectrum via the FLIMJ JavaFX-based user interface for GUI-based analysis and the backing FLIMJ Ops, which expose FLIMLib functionality at the scripting level. The FLIMJ user interface is currently limited to 2D image planes for its calculations, although which plane to analyze is selected by the user via ImageJ's usual time/Z/channel sliders. Extra dimensions can be projected as a sum on selected channels via the ImageJ Ops hyperSliceView function,
   *e.g. “selected_channels = op.transform(). hyperSliceView (hyperdimimage, 3, channels_of_interest)”.* In general, we are strong proponents of good "separation of concerns" in software engineering, meaning that when possible, analysis steps should be kept modular and independent of one another. FLIMLib's integration with ImageJ greatly facilitates this so that users can e.g., apply a spectral unmixing algorithm before or after doing lifetime decay curve fitting. With this design, users have the flexibility to apply steps as needed for their specific data and research, rather than being constrained within one monolithic GUI, which is not extensible beyond its built-in capabilities.
5. A significant number of researchers working on FLIM applications use open source software options such as FLIMfit. How easy/hard would be the transition from FLIMfit to FLIMJ?

   FlimFit is built on MATLAB, whereas FLIMJ is built on ImageJ2. To accommodate MATLAB-based use cases, FLIMLib does include MATLAB wrappers; hence makes it possible to call FLIMLib routines from MATLAB scripts. Integration with ImageJ opens many image processing and analysis tools to users, but if that is not needed, one can choose to use the MATLAB extensions. (<https://github.com/flimlib/flimlib/blob/master/src/matlab/INSTALL.pdf> ).
6. Is FLIMJ including basic FLIM calculations such as optical redox ratio without doing it manually in ImageJ?
   FLIMJ does not handle any other FLIM operations other than basic exponential decay fitting. ImageJ scripts that support multiple scientific languages, including Python, can run image operations, math-operations, statistical operations, and others. Our future directions include directly addressing these metabolic indexing in ImageJ.
